# Two Approaches to Solid-State NMR of Mobile Molecules in Nanoporous Materials

**DOI:** 10.3390/molecules30173603

**Published:** 2025-09-03

**Authors:** Alexander Panich

**Affiliations:** Department of Physics, Ben-Gurion University of the Negev, P.O. Box 653, Beer-Sheva 8410501, Israel; pan@bgu.ac.il

**Keywords:** NMR, intramolecular and intermolecular dipole–dipole interactions, motional averaging, anisotropy, spin–lattice relaxation of dipolar order, spin–spin relaxation, zeolites, intercalation compounds, native collagen fibers, plant stems

## Abstract

This paper reviews two solid-state NMR approaches for investigating mobile molecules in nanoporous materials, with a focus on the motion-averaged dipole–dipole interactions of nuclear spins. The first approach addresses *intramolecular* dipole–dipole interactions, where the anisotropic molecular motion in solids leads to partially averaged interactions that reflect the spatial distribution of molecular positions during motion. The second approach examines *intermolecular* dipole–dipole interactions, which produce anisotropic features in NMR spectra and affect nuclear spin relaxation due to the Brownian motion of molecules within non-spherical nanoscale pores. The applicability of these methods is considered for systems exhibiting molecular mobility, including zeolites, collagen tissues, intercalation compounds, and plant stems.

## 1. Introduction

The study of mobile molecules in nanoporous materials is of considerable interest in both fundamental science and technological applications. Among the many techniques available for investigating such systems, nuclear magnetic resonance (NMR) stands out as a powerful, non-destructive tool for probing molecular dynamics at the atomic scale. In these studies, the primary source of information often comes from anisotropic intramolecular dipole–dipole interactions.

In gases and liquids, these anisotropic interactions are completely averaged out by the rapid, random molecular motion, resulting in sharp and well-resolved NMR lines. However, the complete averaging does not occur for molecules diffusing within the nanopores of crystals, such as water molecules confined in the cavities, channels, or interlayer spaces of zeolites, inclusion and intercalation compounds, and biological tissues, among other materials considered in this work.

In a rigid lattice, the dipolar coupling between two hydrogen nuclei in a water molecule produces a doublet with a frequency separation $\Delta \nu =2\alpha (3\cos^{2}\;\beta -1)$, where *β* is the angle between the proton–proton (p–p) vector and the external magnetic field, and $\alpha =\frac{3}{2}\mu r^{-3}$ [1]. Here, *r* = 1.58 Å is the distance between the hydrogen atoms in a water molecule, and μ is the magnetic moment of the proton. This yields *α* = 22.99 kHz [1], and the maximal splitting at *β* = 0° is 91.95 kHz.

**Figure 1:**
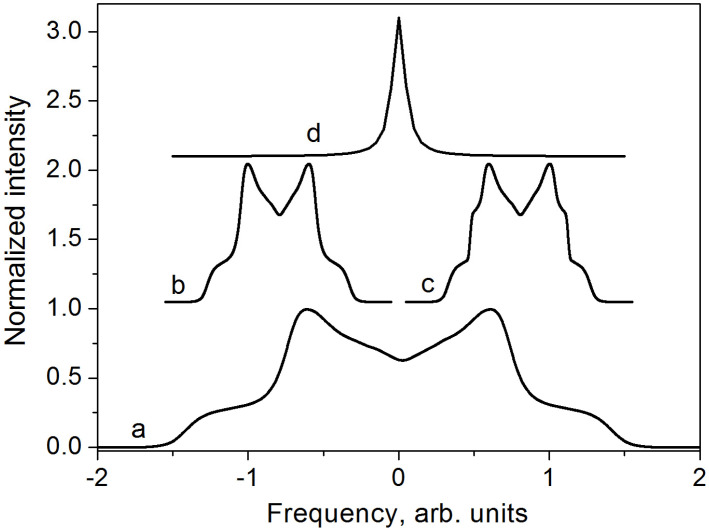
Sketch of ^1^H NMR spectra for water molecules in polycrystalline samples: a—rigid solid; b, c—respectively, axial and non-axial tensors of partially averaged intramolecular dipole–dipole interactions in mobile molecules; d—complete averaging of intramolecular dipole–dipole interactions.

When a water molecule rotates much faster than the dipolar interaction frequency, the angle *β* fluctuates rapidly. In the simplest case, where the molecule rotates around an axis inclined at an angle *θ* to the magnetic field, the dipolar splitting is reduced by a factor of $\frac{1}{2}(3\;\cos^{2}\;\theta -1)$ [2,3,4]. As a result, the dipolar interaction is only partially averaged, and the spectrum consists of a characteristic “narrow doublet” (Figure 1).

This paper reviews two solid-state NMR approaches for investigating molecular mobility in nanoporous materials. The first is based on the partial averaging of *intramolecular* dipole–dipole interactions of nuclear spins in solids due to anisotropic molecular motion. In this case, the averaged interaction reflects the distribution of molecular positions during motion. This approach is well-supported by experimental data and has gained broad acceptance in the scientific community. The second approach examines the emergence of anisotropic features in NMR spectra and nuclear spin relaxation arising from *intermolecular* dipole–dipole interactions during the Brownian motion of molecules within non-spherical nanoscale pores, where intramolecular interactions are fully averaged out. We compare and contrast these two models, discussing their similarities and differences, and discuss their applicability for systems such as zeolites, collagen tissues, intercalation compounds, and plant stems. We show that the approach involving the partial motional averaging of the *intramolecular* dipole–dipole couplings of nuclear spins is well suited for nanoporous materials with a crystalline structure—such as zeolites, fibrous proteins, and intercalation compounds—where molecules occupy specific positions in the crystal lattice and undergo diffusion between these positions. While the approach, based on the Brownian motion of molecules and taking into account only the *intermolecular* dipole–dipole couplings of nuclear spins, implying a complete averaging of intramolecular interactions, is more applicable to some porous structures, preferably amorphous ones, and especially to systems filled with molecules other than water that do not form hydrogen bonds with the host matrix.

## 2. Averaging of Nuclear Dipole–Dipole Interactions of Mobile Molecules in Solid-State NMR

### 2.1. Intramolecular Dipolar Interactions in Mobile Molecules

A detailed theory describing the averaging of the intramolecular dipole–dipole interactions of nuclear spins due to the molecular motion in solids was developed by Gabuda et al. [3,4,5]. As shown in their works, the rapid rotational or diffusional motion of molecules within crystals leads to motion-averaged dipole–dipole interaction tensors. These tensors are determined by both the full set of structural positions occupied by the molecule during diffusion and the spatial symmetry of the host crystal lattice. Within the tensor formalism, the splitting of the ^1^H NMR spectrum of a stationary water molecule due to dipole–dipole interaction is given by Equation (1):

(1) $$D_{kl}=\frac{\gamma^{2}\hbar }{R^{3}}(3e_{k}e_{l}-\delta_{kl}),$$

where $D_{kl}$ is the axially symmetric second-rank dipolar coupling tensor, *R* is the distance between the nuclei, γ is the nuclear gyromagnetic ratio, *e*_k_(*k* = *x*, *y*, *z*), *x*, *y*, and *z* are components of the unit vector pointing from one spin to another, and $\delta_{kl}$ is the Kronecker delta. For a diffusing water molecule, the averaged doublet splitting is given by Equation (2):

(2) $$\Delta \nu =W{\bar{D}}_{zz}(3\cos^{2}\;\theta -1+\eta \sin^{2}\;\theta \cos2\phi )$$

where $W=\frac{3}{4}\gamma$, ${\bar{D}}_{kl}=\sum_{i=1}^{m}p_{i}D_{kl}^{i}$ is the motionally averaged dipole–dipole interaction tensor, *m* is the number of structural positions sampled by the molecule, *p_i_* is the weighting factor satisfying the condition $\sum_{i=1}^{m}p_{i}=1$, *θ* and *φ* are the spherical coordinates of the external magnetic field in the principal axes system of the averaged tensor, and $\eta =({\bar{D}}_{yy}-{\bar{D}}_{xx})/{\bar{D}}_{zz}$ is the asymmetry parameter. Only when the set of positions sampled by the molecule during diffusion exhibits cubic symmetry does the dipole–dipole interaction average completely to zero, resulting in a sharp single line in the NMR spectrum for all orientations. By contrast, trigonal, tetragonal, and hexagonal symmetries lead to an axially symmetric averaged tensor (*η* = 0), producing a characteristic narrow doublet. Triclinic, monoclinic, and orthorhombic symmetries result in a non-axially symmetric averaged tensor (*η* ≠ 0). The corresponding spectra are illustrated in Figure 1.

In the latter two cases, anisotropic diffusion within a restricted geometry leads only to the partial averaging of anisotropic dipole–dipole interactions. The resulting line shapes of diffusing molecules in various compounds have been extensively studied and reported in the literature (see Ref. [4] and the references therein). An analysis of such spectra provides valuable insights into the molecular arrangement and dynamics of the system. For a more comprehensive discussion of this approach, the reader is referred to the excellent review [4], which presents the fundamental principles and key results of Gabuda’s theory on the averaging of local magnetic fields in solid-state NMR studies of atomic and molecular mobility. According to this model, molecular motion leads to the averaging—and consequently, a reduction—of local magnetic fields acting on resonating nuclei, provided that the characteristic frequency of motion *ν_c_* exceeds the NMR line width (*ν_c_* ≥ 10^4^ Hz) [2,3,4,5]. This framework links the local fields to intramolecular nuclear spin interactions and correlates them with structural features and the nature of atomic and molecular motion. It has been successfully applied to a wide range of systems, including ionic and molecular crystals, zeolites, molecular sieves, hydrated proteins, and related materials. The model also accounts for phenomena such as phase transitions in guest subsystems, dynamic disorder, and the influence of correlated electron motion on atomic and molecular mobility and localization. This theory of averaging intramolecular dipole–dipole interactions has been validated in hundreds of experiments across diverse porous crystalline systems containing entrapped mobile molecules, including zeolites, crystal hydrates, intercalation and inclusion compounds, and hydrated proteins [3,4,5]. As a result, this approach has gained broad acceptance within the scientific community.

It is worth noting that, while NMR spectra of diffusing molecules confined within restricted geometries have been extensively studied, the corresponding NMR relaxation processes in such systems remain less thoroughly investigated. In solid insulators containing light spin–1/2 nuclei—such as hydrogen—spin-lattice and spin–spin relaxations are typically governed by dipole–dipole interactions among nuclear spins [6]. The temperature dependence of the relaxation times provides crucial information about molecular dynamics. Furthermore, the secular part of the dipole–dipole interaction, which commutes with the Zeeman term, constitutes an independent energy reservoir [7,8] characterized by its own spin temperature—distinct from that of the Zeeman reservoir. In systems where the dipolar interaction tensor is axially symmetric, both the spin–lattice relaxation rate of the dipolar order and the spin–spin relaxation rate are proportional to the angular factor ${\left(3\cos^{2}\;\theta -1\right)}^{2}$. This factor reflects the orientation of the external magnetic field relative to the principal axis of the averaged dipolar coupling tensor. The doublet splitting Δν and relaxation rates can be expressed as follows [2,9,10,11,12,13,14,15,16,17]:

(3) $$\Delta \nu =D(3\cos^{2}\;\theta -1)$$

(4) $$R_{1D}=R_{1D}^{\#}+R_{1Ddip}{\left(3\;\cos^{2}\;\theta -1\right)}^{2}$$

(5) $$R_{2}=R_{2}^{\#}+R_{2dip}{\left(3\;\cos^{2}\;\theta -1\right)}^{2}$$

where *D* is the constant of the nuclear dipole–dipole interaction, $R_{1Ddip}$ is the spin–lattice relaxation rate of the nuclear dipolar reservoir, $R_{2dip}$ is the spin–spin relaxation rate due to nuclear dipolar coupling, $R_{1,2D}^{\#}$ are the additional isotropic contributions primarily arising from the interaction of nuclear spins with paramagnetic defects and/or impurity ions, and *θ* is the angle between the principal axis of the dipolar interaction tensor and the external magnetic field. The angular factor (3cos *θ*^2^ − 1) = 0 at the angle *θ* = 3cos^2^54.74°.

### 2.2. Intermolecular Dipolar Interactions in Mobile Molecules

**Figure 2:**
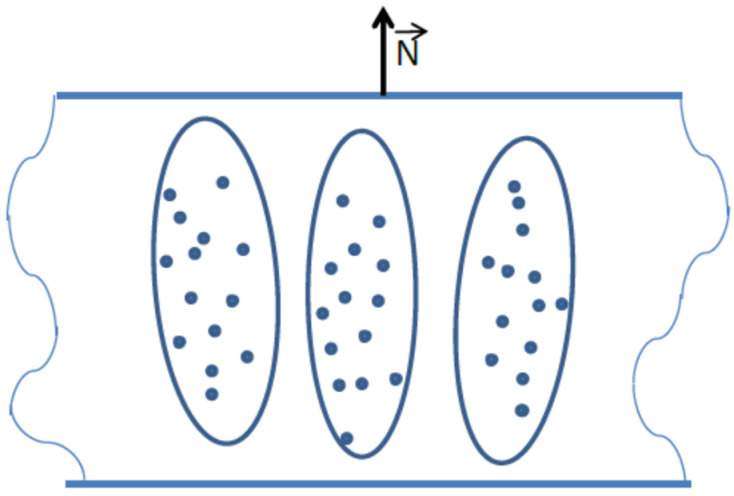
Sketch of a cross-section of a-Si:H thin film with elongated nanovoids containing H_2_ molecules (blue dots). The voids show alignment primarily in the film growth direction (along the film normal **N**).

An alternative approach was pioneered by Baugh et al., who conducted a novel proton NMR study of hydrogenated amorphous silicon (a-Si:H) films containing elongated nanovoids aligned perpendicularly to the film surface (along the growth direction), which host mobile H_2_ molecules (Figure 2) [18,19].

The NMR study of this material revealed no Pake doublet down to 8 K, indicating that the intramolecular dipole–dipole interactions between nuclear spins in hydrogen molecules were averaged out by the random (Brownian) molecular motion. Consequently, the experimentally observed angular dependence of the line width and the nuclear spin–spin relaxation time was attributed to intermolecular dipolar interactions between hydrogen nuclei in molecules confined within a nanoscale volume.

Following the work of Baugh et al., this approach was further extended by Furman et al., who theoretically investigated nuclear relaxation in gas or liquid molecules undergoing Brownian motion within restricted ellipsoidal cavities [20,21,22]. They argued that, while intramolecular interactions are fully averaged out under such conditions, spin–spin and spin–lattice relaxation processes exhibit angular dependence due to *intermolecular* dipole–dipole coupling in cavities smaller than 400 nm [20], 700 nm [22], or 750 nm [21]. They applied this model extensively to interpret the existing NMR data on collagen fibers in biological tissues—including canine articular cartilage, bovine Achilles, and flexor tendons—as well as in plant material, such as celery stalk, and inorganic systems like zeolite vermiculite.

The following sections explore the application of these approaches to the investigation of mobile molecules in zeolites, collagen, and various intercalation and inclusion compounds.

### 2.3. Zeolites

The ability of zeolites to adsorb and desorb various molecules and ions under ambient conditions [23,24,25,26,27,28,29] implies that these species can diffuse through the open pore network. This migration of adsorbed species is central to many key applications of zeolites, including their use as sorbents, molecular sieves, catalysts, and ion-exchange materials [23,24,25,26,27,28,29]. To gain insight into these processes at the nanoscale, it is essential to investigate the dynamics and spatial distribution of adsorbed molecules within the zeolite framework. Numerous NMR studies on the behavior of water molecules in zeolites were summarized in a recent review [4], which focused on the application of Gabuda’s approach to the interpretation of NMR data for these materials. Additionally, more recent NMR investigations—particularly those involving mordenite and vermiculite, which were published after Ref. [4]—are discussed below.

**Figure 3:**
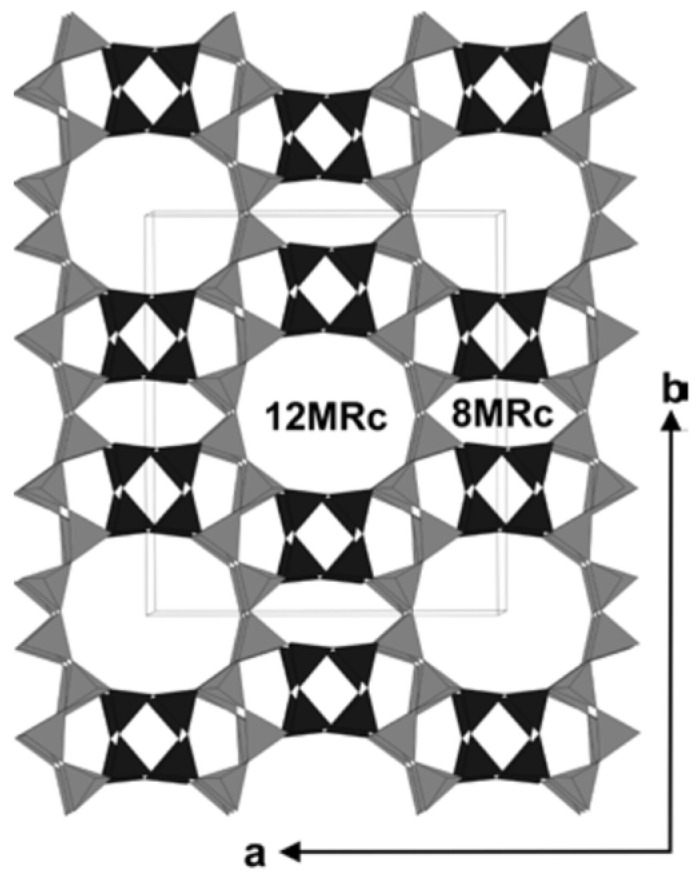
Tetrahedral framework structure of mordenite with unit-cell outlines. The structure is composed of puckered sheets (light gray shading) parallel to (100) formed by six-membered rings of tetrahedra. These sheets are connected along the b-axis by four-membered ring pillars (dark gray shading) in such a way that the 12-membered ring channels (12MRc) and the compressed 8-membered ring channels are formed, both extending along the c-axis that is perpendicular to the figure. (Reprinted with permission from Simoncic and Armbruster [32], Copyright 2005: Elsevier).

**Figure 4:**
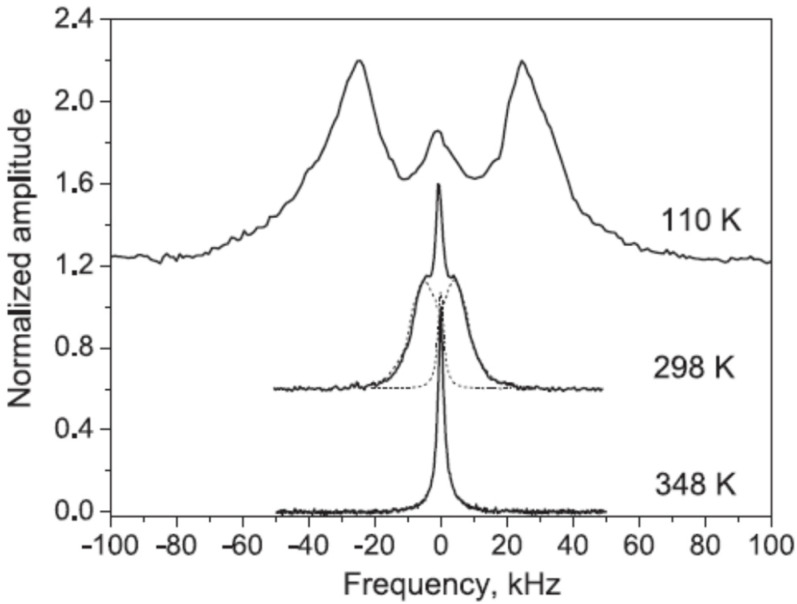
^1^H spectra of powder mordenite sample at different temperatures. Deconvolution of the spectrum at T = 298 K in doublet and singlet components is shown by dashed lines. (Reprinted with permission from Panich et al. [10], Copyright 2016: Elsevier).

The structure of mordenite [30,31,32] consists of straight, ellipsoidal twelve-membered rings (12MRc, aperture 7 × 6.5 Å) and highly compressed eight-membered rings (8MRc, aperture 5.7 × 2.6 Å), which form nanochannels oriented parallel to the *c*-axis (Figure 3). Water molecules are primarily located in the wider 12MRc channels, though they are also present in the narrower 8MRc channels. As a result, the diffusion of larger extra-framework ions and molecules is effectively restricted to one-dimensional pathways. Water diffusion along these channels is observed at temperatures above 200 K [10]. The emergence of a narrow doublet, indicative of molecular diffusion, is shown in Figure 4. In this sample, we were able to measure the nuclear spin–lattice relaxation not only in the laboratory and rotating frames, but also for the dipolar order.

The latter arises from the energy transfer from Zeeman to dipolar order, as discovered by Jeener et al. [7,8], and is observed only due to the partial averaging of the intramolecular dipole–dipole interactions between nuclear spins in water molecules—interactions that remain sufficiently strong to support the formation of a dipolar ordered state [10]. Additionally, we note a contribution from the interaction of nuclear spins with paramagnetic ions, which is characteristic of natural zeolites.

**Figure 5:**
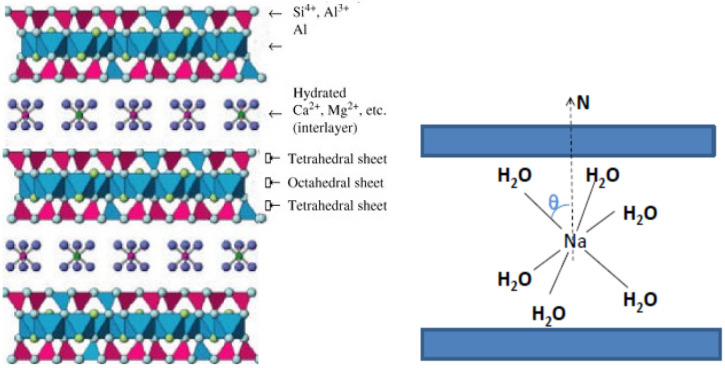
Sketch of the structure of vermiculite (**left panel**) and of the sodium hydrated shell in the interlayer pores of vermiculite (**right panel**). θ is the angle between the octahedron axis of the hydrated shell and the normal to the surface N. Adapted from refs. [11,17], correspondingly. Reprinted with permission from Panich and Baranov [17], Copyright 2025: Elsevier, and from Panich and Swenson [11], Copyright 2024: Elsevier.

Vermiculite clay is a layered material composed of clay platelets separated by one or two molecular layers of water, which form hydration shells around interlayer cations, such as lithium, sodium, magnesium, calcium, and barium (Figure 5). We recently investigated a single crystal of the two-layer hydrate of Na-vermiculite, characterized by a well-defined interlayer spacing of 6.4 Å [11]. Precession X-ray diffraction (XRD) images of this crystal [17] revealed uniform, sharp diffraction spots without streaking or broadening, indicating the absence of significant angular misalignment between the *a–b* planes. This observation confirms that all *a–b* planes—including the surface layer—are coplanar within the resolution limits of the diffraction experiment, attesting to the high quality of the crystal. 

**Figure 6:**
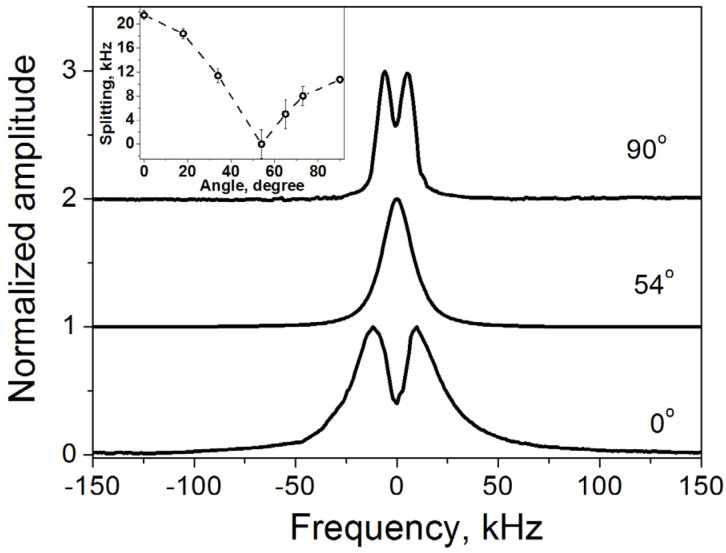
^1^H spectra in a vermiculite single crystal at different angles between the normal to the crystal surface and the applied magnetic field. Angular dependence of the spectral splitting is shown in the inset. (Reprinted with permission from Panich and Swenson [11], Copyright 2024: Elsevier).

**Figure 7:**
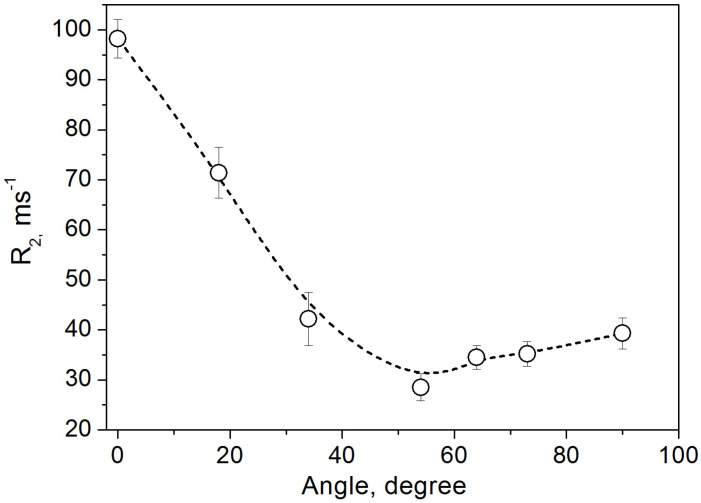
Experimental proton spin–spin relaxation rates *R*_2_ (open circles) in the single crystal of vermiculite at different angles between the applied magnetic field and the normal to the crystal surface. Simulation with the function of Equation (5) is shown by a dotted line. (Reprinted with permission from Panich and Baranov [17], Copyright 2025: Elsevier).

**Figure 8:**
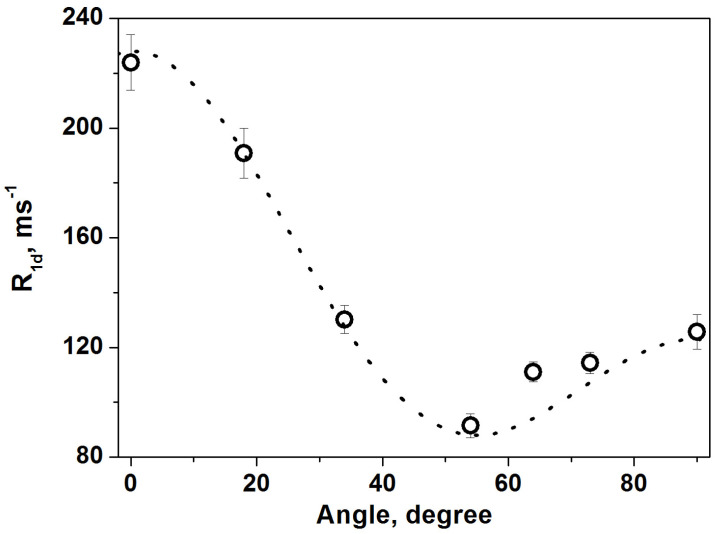
Experimental ^1^H spin–lattice relaxation rate of dipolar order *R*_1*D*_ in vermiculite single crystal at different angles between the applied magnetic field and the normal to the crystal surface (open circles) and calculated curve with the function of Equation (4) (dotted line). (Reprinted with permission from Panich and Swenson [11], Copyright 2024: Elsevier).

The proton NMR spectrum (Figure 6) displays a doublet with angular-dependent splitting, arising from the partially motion-averaged intramolecular dipole–dipole interactions between nuclear spins in the H_2_O molecule. The observed splitting is 10.74 kHz at *θ* = 90° and 21.48 kHz at *θ* = 0°, significantly smaller than those in ‘rigid’ molecules, which exhibit splittings of 45.98 and 91.95 kHz, respectively. Here, θ is the angle between the normal to the crystal surface and the applied magnetic field. Figure 7 and Figure 8 present the experimental angular dependences of the proton spin–spin relaxation rate (*R*_2_) and the spin–lattice relaxation rate of dipolar order (*R*_1D_) in this single crystal [11]. The two-dimensional diffusion of H_2_O molecules between the layers leads to the principal axis of the averaged dipole–dipole interaction tensor being oriented perpendicular to the layers [33,34,35]. Notably, the splitting vanishes, and both relaxation rates reach a minimum at the magic angle of 54.74°, in agreement with expressions (3)–(5). This provides direct evidence that the measured parameters reflect dipole–dipole coupling in a well-aligned single crystal.

**Figure 9:**
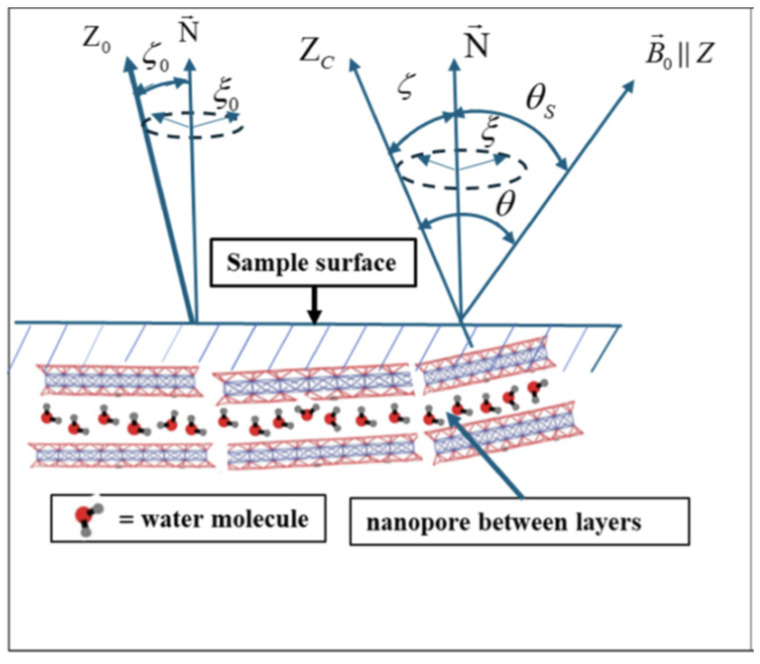
Sketch of nanopores with mobile water molecules in the interlayer space. Here, **N** is the normal to the crystal surface, and ***B*_0_** is the applied magnetic field. (Reprinted with permission from Aptekarev et al. [36], Copyright 2025: Elsevier).

By contrast, the application of the approach [20,21,22] to the study of vermiculite yielded a markedly different structure [36]. Using our experimental NMR data on proton dipolar relaxation in a single crystal of vermiculite [11], Aptekarev et al. [36] performed theoretical calculations within the framework of their model. Based on these calculations, they concluded that the vermiculite crystal is not a single continuous structure, but rather a collection of misaligned crystallites with an average tilt of 0.57 rad (32.7°) relative to the normal to the crystal surface (Figure 9). In their model, water molecules are confined within 2.3 nm-long nanopores. However, our XRD results clearly contradict this interpretation. Contrary to the structure shown in Figure 1 of [36] reproduced in our Figure 9, the vermiculite single crystal does not consist of disconnected particles, nor does it feature 2.6 nm pores or the proposed azimuthal rotation of 1.55 rad (88.8°). Instead, it exhibits extended, continuous interlayer galleries filled with water molecules and sodium ions—features characteristic of natural layered materials and zeolites. These galleries span the entire crystal and are responsible for the well-known sorption, molecular sieving, and ion-exchange properties of such materials [23,24,25,26,27,28,29,30,31,32]. These properties—particularly of zeolites like clinoptilolite—were harnessed in the aftermath of the Chernobyl disaster in 1986 to remove Cs and Sr radioisotopes from contaminated wastewater and drinking water in the Pripyat River, to create protective soil barriers, to reduce cesium levels in milk via livestock feed, and to develop tablets and food additives for human use [37].

In our high-quality vermiculite single crystal, all layers are parallel to each other and to the crystal surface, which corresponds to the outermost layer [17]. It is evident that, if the crystals were composed of crystallites inclined at an average angle of 0.57 rad (32.7°) relative to the surface, as calculated in [36], the minima of the relaxation rates discussed above would occur at an angle significantly different from the magic angle of 54.74°.

The clear contradiction between the calculations presented in [36] and our experimental data stems from the theoretical approach adopted by the authors [36]. At the outset of their paper, they assert that intramolecular nuclear dipole–dipole interactions are fully averaged out in a three-dimensional liquid but remain non-averaged in a two-dimensional liquid. To support this claim, they apply their model of Brownian motion in anisotropic nanocavities [20] to a layered vermiculite crystal. However, this approach contains several errors. It is well established that the averaging of intramolecular dipole–dipole interactions is not determined by dimensionality of the system, but rather by the symmetry of the spatial distribution of molecular positions during motion [3,4,5]. Moreover, Brownian motion—being inherently random—leads to the complete averaging of such interactions. This scenario does not apply to vermiculite, where water molecules do not undergo random Brownian motion. Instead, molecular diffusion occurs along well-defined pathways constrained by the crystal lattice. As a result, intramolecular dipole–dipole interactions are only partially averaged, which accounts for the experimentally observed angular dependence of both the spectral doublet splitting and the nuclear relaxation times.

Let us clarify this issue in more detail. Local magnetic fields at nuclear sites arising from dipole–dipole interactions follow the characteristic angular dependence of (3cos^2^*θ* − 1) [1,2,3,4,5], where *θ* is the angle between the principal axis of the dipolar interaction tensor and the applied magnetic field. Averaging of this interaction occurs due to rapid fluctuations of θ during molecular motion [2,3,4,5,17]. Molecular diffusion consists of both translational and rotational components. In low-dimensional systems, translational motion is restricted and does not contribute to the averaging of intramolecular dipole–dipole interactions, as the angle θ remains unchanged [3,17]. The averaging is instead primarily governed by rapid molecular rotation, which causes rapid fluctuations in *θ* and, consequently, in the local magnetic fields experienced by the nuclei due to dipole–dipole interactions [3,17]. This principle was established by Gabuda et al. several decades ago [3,4,5] and has since been confirmed through numerous experimental studies on various crystals. When the spatial distribution of molecular orientations during motion exhibits cubic symmetry, intramolecular dipolar interactions are completely averaged out. However, for systems with lower symmetry, this averaging is only partial.

This is precisely the case for vermiculite, where the restricted rotational motion of water molecules within the interlayer galleries results in only the partial averaging of the intramolecular dipole–dipole interactions of nuclear spins. Consequently, the NMR spectrum exhibits a characteristic doublet structure, along with an angular dependence of both the splitting and the relaxation rates associated with dipolar coupling. Similar behavior has been reported in a variety of zeolites [3,4,5,10,11,17,28,35,38], for which the theoretical framework proposed in Ref. [36] is entirely inapplicable.

The nature of water in zeolites has been a subject of scientific debates for over a century [23,39,40,41,42]. Various interpretations have been proposed, with water in zeolites being described as capillary or adsorbed water [42], an electrolyte [41], an anisotropic liquid [4], confined water [43,44,45], or two-dimensional water [45,46,47]. This discourse remains ongoing. Nonetheless, while water molecules in the channels and interlayer spaces of zeolites are sometimes loosely described as “liquid”, they do not exhibit the characteristics of a true liquid state. A genuine liquid is defined by the absence of long-range order and the presence of random fluctuations in intermolecular force fields. However, decades-old X-ray, neutron diffraction, and NMR studies have demonstrated that H_2_O molecules in most natural zeolites occupy well-defined crystallographic positions [48,49,50,51,52,53,54,55,56,57,58,59], with their orientations and proton–proton vectors also being precisely defined within the zeolite framework. Likewise, cations are located at specific sites within the zeolite pores to compensate for the negative charge of the $AlO_{4}^{-}$ tetrahedra [28,51,53,55]. The mobility of both water molecules and cations occurs as jumps between these fixed lattice positions, rather than via Brownian motion.

The translational diffusion coefficient of water, *D*, measured in two-H_2_O layer Na-vermiculite clay using quasi-elastic neutron scattering at T = 295 K [47], was found to be *D =* 8.8 × 10^−10^ m^2^/s, indicating dynamics that are 2.5–3 times slower than those of bulk water, which has *D =* 2.5 × 10^−9^ m^2^/s [60] or *D* = 2.2 × 10^−9^ m^2^/s [61]. A similar diffusion coefficient, *D =* 7 × 10^−10^ m^2^/s, was reported for the two-H_2_O layer Li-montmorillonite clay [60,62].

It is important to note that a dipolar ordered state of water molecules has been experimentally observed in several zeolites [10,11,63], which, like vermiculite, do not represent a two-dimensional liquid. This behavior is characteristic of the vast majority of zeolites. Additionally, natural zeolites typically contain paramagnetic ions, whose interactions with nuclear spins dominate the nuclear spin-lattice relaxation processes, significantly outweighing the contributions from nuclear dipole–dipole interactions [2,10,11,28]. Neglecting this contribution, as in [36] can lead to erroneous conclusions.

In summary, all ^1^H NMR data on confined water molecules in zeolites are accurately described by the model of partial motional averaging of local magnetic fields induced by nuclear dipole–dipole interactions [3,4,5]. Models based on Brownian motion or layer misalignment are inappropriate for such systems.

### 2.4. Native Collagen Fibers

**Figure 10:**
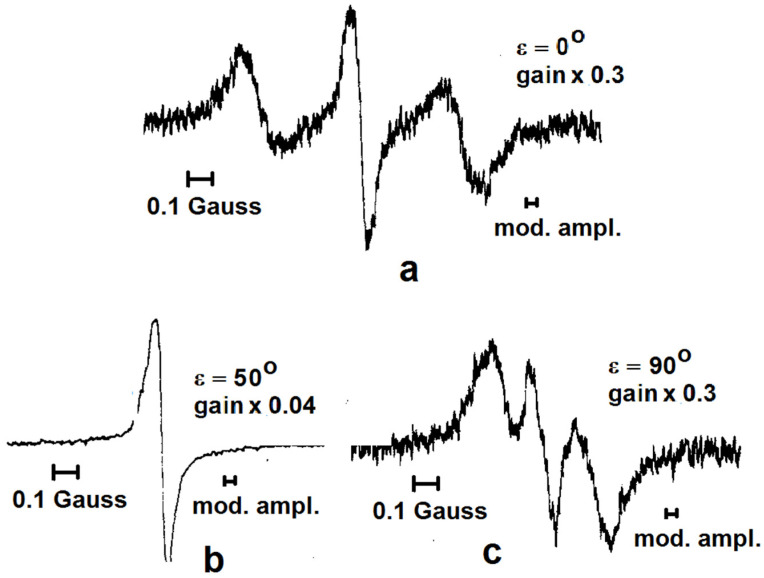
Proton magnetic resonance curves from the water in hydrated collagen (oriented rat-tail tendon) containing 36 g water per 100 g dry weight (relative humidity 81%). The curves show the derivative of the resonance signal at angles ε of 0° (**a**), 50° (**b**), and 90° (**c**) between the fiber axis and the applied magnetic field. (Reprinted with permission from Berendsen and Migchelsen [66], Copyright 1965: John Wiley and Sons).

Collagen fibrils are arranged in dense bundles aligned parallel to the tendon axis. In structures such as the Achilles tendon and ligaments, these fibrils exhibit a high degree of order; whereas, in articular cartilage, their orientation is somewhat more random. Gabuda’s approach, which accounts for the partial motional averaging of local magnetic fields in solid-state NMR due to molecular mobility, has also proven effective for analyzing the NMR spectra of water molecules in collagen [4,64]. These spectra display doublets arising from partially averaged dipole–dipole interactions between protons within the water molecule (Figure 10). This phenomenon was first observed by Berendsen in 1962 [65].

Given that collagen molecules are packed with tetragonal symmetry, and the overall structure possesses a fourth-order symmetry axis, Gabuda’s theory predicts that the local magnetic field experienced by the water protons should exhibit axial symmetry, reflecting the symmetry of the sample. The observed splitting of the narrow doublet is consistent with this model, with angular dependence described by the standard dipolar splitting formula $\alpha (3\;\cos^{2}\;\theta -1)$. This behavior has been experimentally confirmed for both normal and heavy water in collagen [4,12,64,65,66,67,68].

It is believed that hydrated collagen contains three types of water molecules [4,64,69,70,71,72]. The first type comprises water molecules located between the triple helices—specifically, between the three α-chains and between adjacent helices within a microfibril. These are tightly bound to the protein surface, forming hydrogen bonds (O–H···O and O–H···N) with the host matrix. This “bound water” plays a crucial role in stabilizing the triple-helical structure and maintaining the thermodynamic integrity and functionality of the collagen molecule. Due to their relatively fixed positions and strong interactions with the crystal structure, these water molecules produce a characteristic doublet in the NMR spectrum, resulting from partially—but not fully—motion-averaged intramolecular dipole–dipole interactions. The second and third layers of water, positioned beyond the directly bound layer, are also influenced by active sites on the protein surface and contribute to the local magnetic field, albeit to a lesser extent. The remaining water in well-hydrated collagen tissue is considered “loosely bound water”. Its structure is sometimes thought to resemble that of disordered ice [4,64].

All of these water molecules exhibit anisotropic motion modulated by the architecture of the collagen matrix. Furthermore, dynamic exchange processes occur between the different types of water molecules. The combination of constrained motion and molecular exchange results in the partial averaging of the dipolar interactions, producing the spectral features observed experimentally [4,12,64,65,66,67,68]. The presence of a doublet in the NMR spectra indicates that water motion in native collagen is not entirely random (i.e., not Brownian) but displays a certain degree of order. The average local field constant *α* varies among species, with values of 0.49, 0.51, and 0.68 kHz reported for human, bovine, and rat collagen, respectively [64].

We emphasize that the observed doublet splitting arises from partially averaged, angle-dependent *intramolecular* dipolar interactions between the protons of water molecules. These interactions clearly contribute to the angular dependence of both proton spin–spin (*T*_2_) and spin–lattice of dipolar order (*T*_1D_) relaxation rates. However, this key factor was overlooked in the aforementioned studies [20,21,22], in which the angular dependences of nuclear spin relaxation rates in collagen were analyzed exclusively in the framework of intermolecular dipole interactions, based on a model assuming random (Brownian) motion of molecules.

As demonstrated above, water molecules in collagen do not undergo random Brownian motion. Instead, their movement is constrained to specific positions within the crystal lattice. If Brownian motion were present, it would lead to the complete averaging of the intramolecular dipole–dipole interactions—contradicting experimental observations, which consistently show only partial averaging.

Navon et al. [12], whose experimental data were used by Furman et al. in Ref. [21], explicitly stated that they investigated the angular dependence of *intramolecular* dipolar interactions, not the *intermolecular* ones mentioned in [21]. The conclusions [21], based solely on fitting theoretical models to Navon et al.’s data, therefore rest on a fundamental misinterpretation of the original experimental context. Similarly, an examination of the studies by Du et al. [73] and Shao et al. [74], whose data on spin–lattice relaxation in the rotating frame (*T*_1ρ_) and spin–spin relaxation were used by Furman et al. [75], suggests that these works analyzed the contribution of residual intramolecular dipolar interactions—those arising from the restricted anisotropic motion of water molecules within the collagen matrix.

It is widely acknowledged that the anisotropy of *T*_2_ relaxation in collagen-rich tissues, such as cartilage and tendon, is due to non-zero residual *intramolecular* dipolar couplings, resulting from the preferential orientation of water molecules bound to the aligned collagen fibrils [76]. These residual couplings, characteristic of “bound” water molecules, are the dominant mechanism behind transverse relaxation anisotropy in such systems [76].

In experimental studies [77,78,79,80,81,82,83], the intra- and intermolecular contributions to water proton relaxation were generally not separated; thus, the measured relaxation likely reflects a combined effect of both. However, earlier analyses by Berendsen [65] and Gabuda and Rzhavin [64] indicate that the magnitude of residual intramolecular dipolar interactions is typically several times greater than that of intermolecular interactions. Thus, theoretical approaches that consider only intermolecular dipole contributions, such as those employed in [20,21,22], are insufficient for interpreting experimental data on collagen and lack significant scientific validity.

A notable exception is the work by Navon et al. [12], who employed an in-phase double quantum filtered (IP-DQF) pulse sequence to separate the water signal from the protein signal, exploiting the differences in their intramolecular dipolar interactions. This method enables selective excitation of either water or protein signals. However, as noted above, Navon et al. focused specifically on intramolecular dipolar interactions and their angular dependence.

Moreover, the ^1^H NMR spectrum of collagen consists of two components: a narrow doublet arising from mobile water molecules and a broad line originating from the protein matrix. The broad component also exhibits angular dependence—for example, ranging from 25.5 to 38.3 kHz as the angle between the direction of collagen fibers and the external magnetic field varies from 90° to 0° in the Achilles tendon of a three-year-old bull [84]. In this tissue, the ratio of hydrogen atoms in protein molecules to those in water molecules is approximately 1:3, suggesting a substantial contribution of dipolar relaxation from the highly oriented collagen fibers.

Fung and Puon [85] reported that this protein-associated component exhibits a very short *T*_2_ relaxation time (~24 µs), which is undetectable in conventional MRI but is clearly observed in solid-state NMR as part of the rapidly decaying initial signal.

By contrast, the proton spin–spin relaxation times *T*_2_ for mobile water molecules in collagen typically range from a few milliseconds to a few hundred milliseconds (e.g., 2–5 ms and 20–90 ms, as shown in [80]). We note that in the above NMR studies [77,78,79,80,81,82,83], the intra- and intermolecular contributions of water protons, as well as the contribution of collagen macromolecular protons to relaxation processes, were not separated, meaning that all components contributed to the angular dependence of the relaxation. The failure of the authors [20,21,22] to take into account the intramolecular dipolar interactions of water protons and macromolecular matrix protons makes their model unsuitable for the analysis of NMR data in native collagen-rich tissues.

Moreover, as noted by Momot [16], “neither prediction is consistent with the extensive body of experimental evidence in articular cartilage, where the anisotropic component of 1/*T*_2_ has been convincingly demonstrated to scale as (1 − 3 cos^2^θ)^2^ rather than |1 − 3 cos^2^θ|” (proposed by Furman et al.), “and no appreciable anisotropy has been reported for 1/*T*_1_.” In addition, an extensive critique of Furman’s approach has just been published by Pang et al. [86]. The authors confirmed that it is “well established—both theoretically and experimentally—that transverse relaxation anisotropy in cartilage and tendon primarily originates from residual *intramolecular* dipolar coupling between the two protons in water molecules”, and that “based on current theoretical understanding and experimental evidence, it is unlikely that cartilage nanostructures can be reliably characterized through anisotropic relaxometry of water protons”.

In summary, the doublet structure of proton spectra, caused by intramolecular dipolar interaction, rejects the Brownian motion of water molecules in native collagen—the central assumption of the theoretical model of Furman et al. Therefore, the relevance of this model for NMR relaxation in collagen appears to be unfounded. As Pang et al. [86] concluded, “the claim of significant progress in understanding cartilage ultrastructure presented by Furman et al. appears premature and should be interpreted with caution”.

### 2.5. Intercalation and Inclusion Compounds

**Figure 11:**
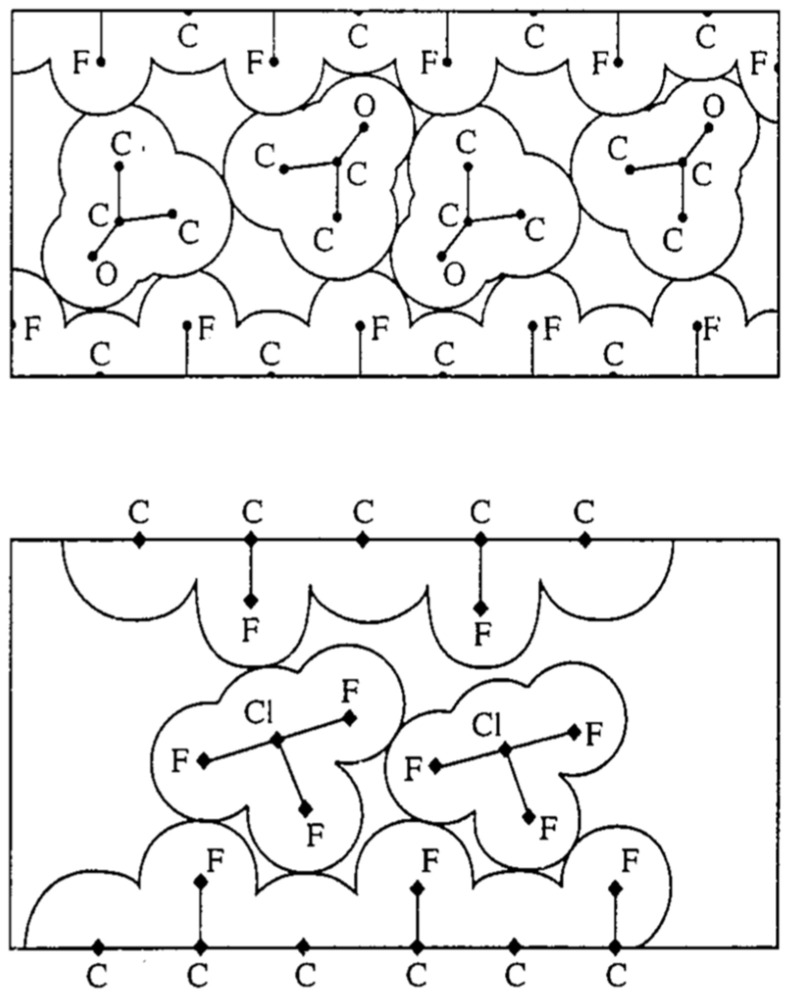
Sketch of the structure of fluorinated graphite intercalated with acetone (**top**) and chlorine trifluoride (**bottom**). (Reprinted with permission from Panich [38], Copyright 1996: Elsevier).

**Figure 12:**
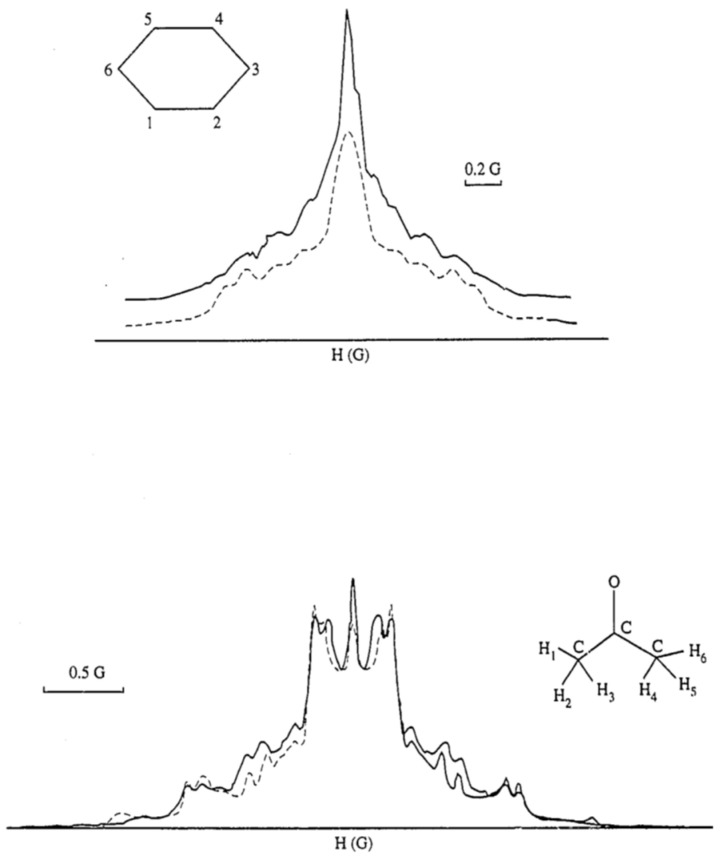
Room temperature ^1^H spectra of benzene (**top**) and acetone (**bottom**) molecules diffusing in the galleries of fluorinated graphite (continuous lines) and the simulated spectra (dashed lines). (Reprinted with permission from Panich [38], Copyright 1996: Elsevier).

The model of partial averaging of intramolecular dipolar interactions provides an effective framework for analyzing the NMR spectra of mobile molecules in intercalation compounds of fluorinated graphite, C_2_F [33,34,35,38,87,88,89,90,91,92,93,94]. The structures of several representative compounds are shown in Figure 11. Figure 12 presents the proton spectra of acetone and benzene molecules diffusing within the interlayer galleries of fluorinated graphite. These molecules contain six spins, resulting in complex spectra that are determined by intramolecular dipole–dipole interactions, which are only partially averaged by two-dimensional diffusion. For instance, in the case of a fixed methyl group, the doublet splitting constant is approximately 16 kHz, but it is reduced by half for a rotating group. For diffusing acetone and acetonitrile molecules, the splitting constants were measured to be 1.9 kHz and 1.36 kHz, respectively.

**Figure 13:**
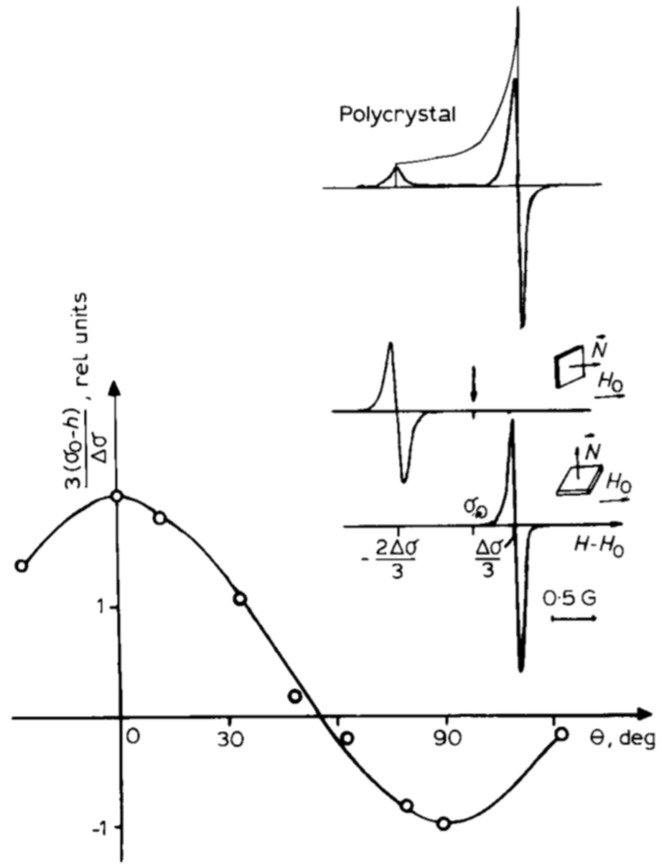
Angular dependence of the narrow component of the ^19^F spectrum in the stage 1 of highly-oriented BrF_3_-fluorine graphite intercalation compound, and the ^19^F spectra (first derivative of absorption line) of this component for two orientations and in a polycrystalline sample. The arrow indicates the position of the signal of the BrF_3_ liquid standard; θ is the angle between the normal *N* to the sample surface and the applied magnetic field *H*_0_. (Reprinted with permission from Panich [91], Copyright 1993: Elsevier).

**Figure 14:**
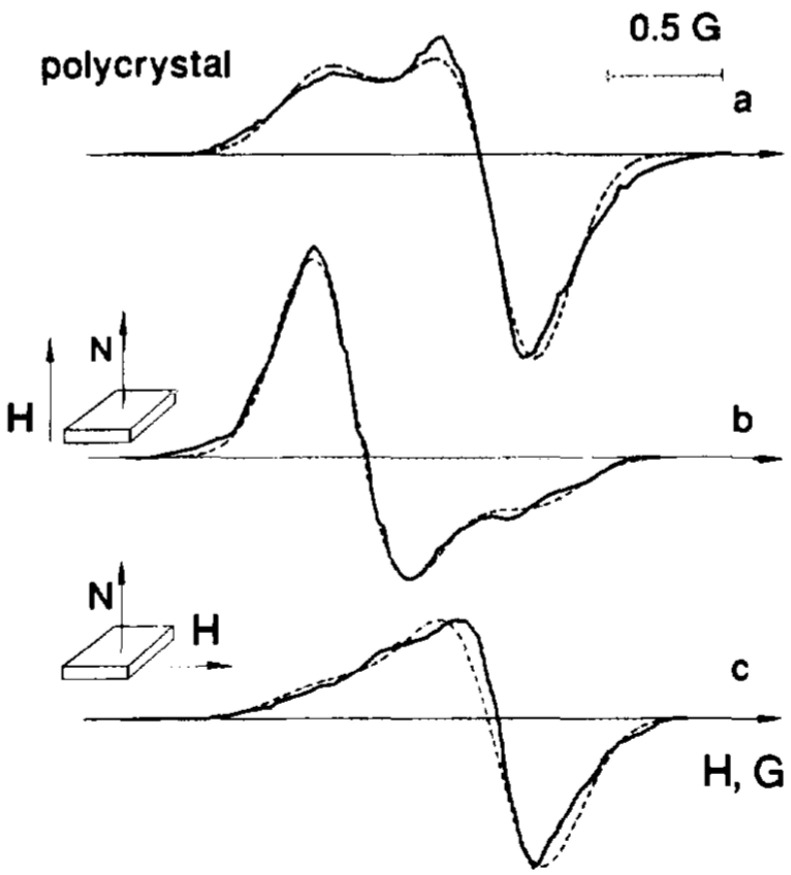
Experimental ^19^F spectra (first derivative of absorption line) of a ClF_3_ molecule in polycrystalline and oriented samples of C_2_F·ClF_3_ at room temperature (continuous lines) and simulated spectra (dashed lines) for chemical shielding anisotropy <Δσ> = −107 ppm. Here, N is the normal to the tablet surface, and H is the external magnetic field. **a**—spectrum of a powder sample, **b** and **c**—spectra of partially oriented samples with an external magnetic field parallel and perpendicular to the normal to the tablet surface, respectively. (Reprinted with permission from Panich [92], Copyright 1994: Elsevier).

J. Baugh’s approach [18,19] has not yet been applied to intercalation compounds in this context. However, it may probably be effective, particularly for bromine and chlorine trifluoride molecules intercalated into a fluorinated graphite matrix [34,35,38,88,91,92,94]. In such systems, intramolecular dipolar interactions of the ^19^F nuclear spins are averaged out by (i) rapid anisotropic molecular diffusion and (ii) intra- and intermolecular chemical exchange between fluorine atoms at ambient temperature. These molecules do not form covalent bonds with the C_2_F matrix; their motion is constrained solely by spatial restrictions. As a result, their ^19^F NMR spectra are governed by chemical shielding anisotropy that is only partially averaged by anisotropic diffusion between the fluorinated graphite layers. The spectra exhibit distinct angular dependencies (Figure 13 and Figure 14), observed using partially oriented samples prepared by applying pressure [33,34,35,38,87,88,89,90,91,92,93,94]. By subtracting the contribution of chemical shielding anisotropy from the overall relaxation, it is possible to separate the contribution of intermolecular dipole–dipole coupling to the relaxation of ^19^F spins. For example, this should work in the first stage of C_2_F·BrF_3_, where different line widths at various orientations in the magnetic field relative to the tablet surface are observed due to intermolecular dipole–dipole interactions of ^19^F spins (Figure 13). Notably, some inclusion compounds form incommensurate phases, which are of particular scientific interest [95,96,97,98].

**Figure 15:**
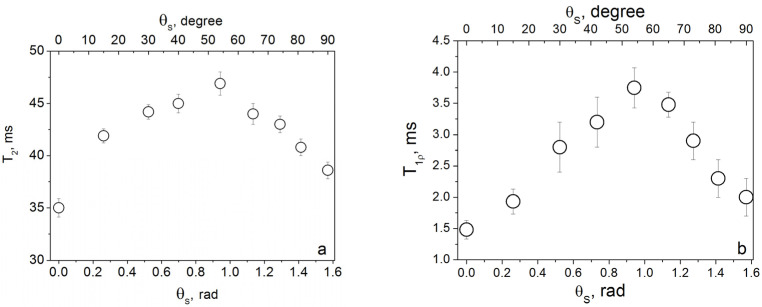
Angular dependences of (**a**) the spin–spin relaxation rate and (**b**) the spin–lattice relaxation rate in the rotating frame of hydrogen molecules in the a-Si:H films [102].

The approach developed by Baugh et al. [18,19] focuses on the NMR study of molecules confined in elongated nanovoids (Figure 2). In this model, intramolecular dipole–dipole interactions of nuclear spins are averaged out by random molecular motion, while the observed anisotropy in line position and relaxation times arises from intermolecular interactions. This model was initially experimentally demonstrated in hydrogenated amorphous silicon (a-Si:H) films [18,19], and was later extended theoretically by Furman et al., who analyzed nuclear spin–spin and spin–lattice relaxation in gas or liquid molecules undergoing Brownian motion within restricted ellipsoidal cavities [20,21,22]. Experimental support for this model also came from further studies of a-Si:H films [99,100,101]. The absence of a doublet structure in the ^1^H spectra indicates the effective averaging of intramolecular dipole–dipole interactions between proton spins. In experiments, anisotropies of both spin–spin and spin–lattice relaxation in the rotating frame were observed (Figure 15) [99,100,101,102], as well as some anisotropy in the position of the resonance line [99].

The observed anisotropy arises solely from intermolecular dipolar interactions between hydrogen spins. Unlike water molecules in zeolites or collagen (discussed below), hydrogen molecules in this material do not form hydrogen bonds with the host matrix, nor do they occupy specific sites within the crystal lattice. As a result, they undergo random Brownian motion within the amorphous film.

Similar behavior is found in certain inclusion compounds, which are solids composed of weakly interacting host and guest molecules. The guest species often exhibit high rotational and translational mobility at ambient temperature. For example, periodic mesoporous silicas, such as MCM-41 (Mobil Composition of Matter No. 41) and SBA-15 (Santa Barbara Amorphous), along with many of their derivatives, feature narrow pore-size distributions and large specific surface areas [103,104,105,106,107,108,109,110,111]. These materials hold significant potential for molecular encapsulation [103,104,105,106,107,108,109,110,111]. Furthermore, they provide an ideal model system for investigating the physicochemical properties of fluid guest molecules confined within porous environments, where solid–liquid and liquid–liquid interactions may strongly compete [109]. The structure and dynamics of such confined guest molecules—revealed through solid-state NMR, relaxometry, molecular dynamics simulations, and calorimetry—have been recently reviewed [112].

### 2.6. Plant Stem

The above approach, considering only the intermolecular interaction of nuclear spins, was also applied to explain the anisotropy of spin–lattice and spin–spin relaxation in celery stems [113,114]. In the latter study [114], the authors repeatedly attributed the angular dependence of relaxation times to mobile water molecules confined within the fibrils. However, in celery, fibrous tissue constitutes only about 2.5% of the total material. The NMR signal from such a small fraction is indistinguishable from the much stronger signals originating from organic tissues, mineral solutions transported from the roots to the leaves, and sucrose solutions moving in the opposite direction. In practice, the NMR signal is primarily recorded from these larger, more abundant regions of the stem, rather than from the fibrils.

The vascular channels through which minerals and sucrose flow have diameters ranging from 16 to 220 μm [115,116,117]—one to two orders of magnitude larger than the sub-400–700–750 nm diameter channels required for the angular dependence of relaxation times (*T*_1_ and *T*_2_) predicted by theory [20,21,22]. Furthermore, in natural celery, relaxation processes are influenced by paramagnetic impurities absorbed from the soil, further complicating the relaxation mechanisms. Thus, the application of the above theory [20,21,22] in this context appears unsubstantiated. Although the celery stalk can be considered as a quasi-one-dimensional system (albeit with structural disorder), leading to some angular dependence of the nuclear spin relaxation times, this phenomenon is not related to the theoretical calculations of fibril behavior performed in [114].

## 3. Conclusions

The approach based on the partial averaging of *intramolecular* dipole–dipole interactions of nuclear spins through molecular motion is particularly suitable for nanoporous materials with crystalline structure—such as zeolites, fibrous proteins, and intercalation compounds—where molecules occupy well-defined lattice positions and diffuse between them [3,4,5,10,11,12,13,14,15,16,17,18,19,28,29,33,34,35,42,43,53,55,56,57,58,59,63,68,73,74,76,77,78,79,80,81,82,83,84,85,86,87,88,89,90,91,92,93,94].

By contrast, the approach that relies on *intermolecular* dipole–dipole interactions, with the complete averaging of intramolecular couplings due to Brownian motion, is better suited for amorphous porous systems, especially those filled with non-aqueous molecules that do not form hydrogen bonds with the host matrix. The absence of such bonds enables more chaotic molecular dynamics [18,19,99,100,101,118,119,120]. Another promising application of this approach is micro- and nanochannels engineered within various matrices. These systems, central to the rapidly growing field of micro- and nanofluidics, provide a platform for probing viscosity and molecular dynamics at small scales [121,122,123,124,125,126,127,128,129,130,131,132,133] and may offer favorable conditions for testing and extending the predictions of this model.

## Data Availability

No new data were created or analyzed in this study. Data sharing is not applicable to this article.
